# Changes in the Organization of Excitation-Contraction Coupling Structures in Failing Human Heart

**DOI:** 10.1371/journal.pone.0017901

**Published:** 2011-03-09

**Authors:** David J. Crossman, Peter R. Ruygrok, Christian Soeller, Mark B. Cannell

**Affiliations:** 1 Department of Physiology, Faculty of Medicine and Health Sciences, University of Auckland, Auckland, New Zealand; 2 Auckland City Hospital, Auckland, New Zealand; University of Cincinnati, United States of America

## Abstract

**Background:**

The cardiac myocyte t-tubular system ensures rapid, uniform cell activation and several experimental lines of evidence suggest changes in the t-tubular system and associated excitation-contraction coupling proteins may occur in heart failure.

**Methods and Results:**

The organization of t-tubules, L-type calcium channels (DHPRs), ryanodine receptors (RyRs) and contractile machinery were examined in fixed ventricular tissue samples from both normal and failing hearts (idiopathic (non-ischemic) dilated cardiomyopathy) using high resolution fluorescent imaging. Wheat germ agglutinin (WGA), Na-Ca exchanger, DHPR and caveolin-3 labels revealed a shift from a predominantly transverse orientation to oblique and axial directions in failing myocytes. In failure, dilation of peripheral t-tubules occurred and a change in the extent of protein glycosylation was evident. There was no change in the fractional area occupied by myofilaments (labeled with phalloidin) but there was a small reduction in the number of RyR clusters per unit area. The general relationship between DHPRs and RyR was not changed and RyR labeling overlapped with 51±3% of DHPR labeling in normal hearts. In longitudinal (but not transverse) sections there was an ∼30% reduction in the degree of colocalization between DHPRs and RyRs as measured by Pearson's correlation coefficient in failing hearts.

**Conclusions:**

The results show that extensive remodelling of the t-tubular network and associated excitation-contraction coupling proteins occurs in failing human heart. These changes may contribute to abnormal calcium handling in heart failure. The general organization of the t-system and changes observed in failure samples have subtle differences to some animal models although the general direction of changes are generally similar.

## Introduction

Excitation-contraction coupling (ECC) in cardiac ventricular muscle occurs via the calcium induced calcium release (CICR) mechanism (for review see [1]). In CICR, the action potential opens L-type calcium channels (dihydropyridine receptors -DHPRs) in the surface membrane and t-tubular membranes to activate the sarcoplasmic reticulum (SR) ryanodine receptors (RyRs) to cause release of Ca^2+^ from the SR. The SR release forms Ca^2+^sparks which summate to produce the cell-wide increase in Ca^2+^ which regulates force [2]. The close and precise alignment of DHPRs and RYRs in a structure called the “couplon” is thought to be critical to efficient CICR [3], [4].

The transverse tubular system (t-tubules) of cardiac ventricular myocytes enables highly synchronized calcium release by CICR at couplons [5]. The t-tubules form a network of plasma membrane invaginations [6], [7], [8] and in human ventricular myocytes, couplons have a transverse spacing of ∼0.8 µm [9]. In a dog model of heart failure, a reduction in t-tubule density was observed [10], [11] and similar alterations have been reported in other heart failure animal models (see below). It is unclear whether these alterations in t-tubule density result in a reduction in couplon density, although such an effect might help explain reduced contractility in heart failure. The idea that microscopic structural alterations in the organization of DHPRs and RyRs within the couplon might play a role in heart failure was first raised in a computer modelling study [12] and this idea has gained traction from rodent studies that have detected a reduced ability of the L-type Ca^2+^ current to trigger CICR [13], [14]. Dyssynchronous or non-uniform CICR associated with a change in t-tubule organisation has also been observed in murine [15], canine [16] and porcine models [17] of heart failure.

Ca^2+^ handling abnormalities have been described in various forms of human heart failure including idiopathic dilated cardiomyopathy (IDCM) [18], [19], [20]. Despite the importance of the t-system in calcium handling, few studies have examined the organization of the t-system in normal and failing human myocardium [21] although t-tubule-related structures are known to be changed [22], [23]. Recently the work of Lyon *et-al* (2009) using scanning ion conductance microscopy and confocal imaging of di-8-ANNEPs labeling suggested a loss of t-system structure in isolated failing human cardiomyocytes [24]. In contrast, the work of Ohler *et-al* (2009) showed no significant change in the structure of t-system between isolated normal and failing cardiomyocytes by using 2-photon imaging of di-8-ANNEPs labeling [25]. A potential limitation in using isolated myocytes is those cells most affected by the disease process may be preferentially destroyed during the isolation procedure, introducing a selective bias into the cells that are analyzed [25].

To gain greater insight into the possible changes in t-system structure and changes in EC coupling proteins, we have used intact tissue and immunohistochemistry combined with high resolution confocal microscopy and quantitative analysis of labelling patterns in ventricular muscle from both healthy human hearts and from patients with idiopathic (non-ischemic) dilated cardiomyopathy (IDCM). This avoids the potential for a selection bias due to cell isolation but also has the advantage that multiple components of the t-system can be labelled. Importantly it also allows a detail assessment of DHPR and RyR labelling, for which no data on the human heart currently exists. Our results demonstrate that disarrangement in ECC structures occurs in human heart failure. The results underpin related animal studies of heart failure but also reveal some unexpected differences in the geometric organization of key structures and proteins in the normal and diseased human heart.

## Methods

### Myocardial tissue

**Figure 1:**
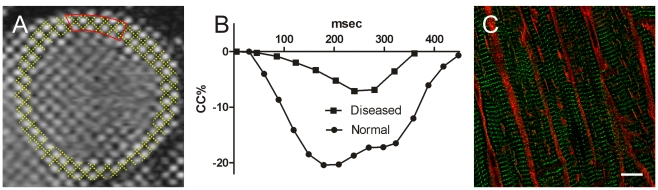
Panel A shows tagged MRI short axis image through the middle of a failing heart in end diastole. Tracked grids are shown as yellow overlay with sampled region marked in red. Panel B shows circumferential shortening (CC%) of the region indicated in the panel A compared to a similar region in normal human heart. Panel C shows RyRs (green) and WGA (red) labeling of diseased myocytes from the region shown in panel A. Image is z projections of 10 slices with z depth of 2.5 µm from deconvolved image stack. Scale bar is 10 µm.

Human cardiac tissue was obtained with the written informed consent of heart transplant recipients and from families of organ donors for normal tissue as approved by the New Zealand Health and Disability Ethics Committee (NTY/05/08/050). Normal tissue samples were obtained from 4 donors that could not be matched to recipients they had a mean age of 60 (54–68), and had normal echo and ECG. Medications for donors included inotropic support in intensive care unit, and one patient was recieving felodipine. Diseased tissue came from 7 end-stage failing hearts from patients diagnosed with idiopathic dilated cardiomyopathy which were class III or IV on the New York Heart Association heart failure scale. These patients had a mean age of 33 years (16–56), mean ejection fraction (by echo) of 17% (5–30), mean heart rate of 84 bpm (70–100), and mean systolic blood pressure 95 mm Hg (90–105). Common drug therapies included renin-angiontensin-aldosterone system modulators (cilazapril, spironolactone, quinapril, losartan), diuretics (furosemide, bendroflumethiazide), beta blockers (carvedilol, metoprolol) and anticoagulants (warfrin, asprin). Other cardiac medications included digitalis, amiodarone, simvastatin, and dobutamine. Reduced contraction strength in three failing hearts was confirmed by tagged MRI and tissue was obtained directly from the operating theatre (e.g. Figure 1). Samples were taken from the midline region of the left ventricle, fixed with 1% paraformaldehyde in PBS overnight at 4°C, cryoprotected in 30% sucrose, frozen with liquid nitrogen chilled isopentane and stored at −80°C until further processing. 30 µm thick frozen sections were cut on a Leica CM 1900 cryostat and mounted on either glass slides or coverslips for subsequent antibody labelling.

### Indirect immunofluoresence labeling

Labelling with wheat germ agglutinin (WGA) was carried out by hydrating and incubating with WGA conjugated to Alexa Fluor 594 (Molecular Probes/Invitrogen). Double labelling for membrane proteins and WGA included blocking with FX signal enhancer (Molecular Probes/Invitrogen) for 1 hour. The sections were then incubated with the following antibodies: dihydropyridine receptor (DHPR, ACC-003 (a Cav 1.2 II-III loop specific antibody shown here) and ACC-013 (raised against Cav 1.2 N terminal residues 1-46 see supplementary Figure S1), Alomone Labs), ryanodine receptor 2 (RyR, MA3-916, Affinity Bioreagents), sodium calcium exchanger (NCX, AB2869, Abcam) or caveolin-3 (Cav-3, AB2912, Abcam) overnight at 4°C. Sections were washed three times in PBS and incubated with secondary antibodies labelled with Alexa Fluor 488 and wheat germ agglutinin Alexa Fluor 594 (Molecular Probes/Invitrogen). *f*-actin labelling followed the protocol for double labelling of membrane proteins and WGA, except WGA was replaced with phalloidin conjugated to Alexa Fluor 594 (Molecular Probes/Invitrogen). Controls consisted of omitting primary antibodies, WGA and phalloidin to check for non-specific labelling of secondaries and cross-talk. For DHPR labelling, the specificity of antibody was further tested by pre-incubation with the supplied blocking peptide (Cav1.2_848-865_ peptide) and running a Western blot against human cardiac tissue homogenate which gave major band at 240 kda and minor band 100 kDa, confirming results provided in the manufacturer's data sheet.

### Imaging

Fluorescent images of single and double labelled tissue sections were recorded with a Zeiss LSM410 confocal microscope using a Zeiss 63x NA 1.25 oil-immersion objective. Images of tripled labelled tissue sections were recorded with a Olympus FV1000 confocal microscope using a Olympus 60x NA 1.35 oil-immersion objective. Three-dimensional images stacks were acquired (with lateral and axial pixel spacings of 0.1 µm and 0.2 µm respectively).

### T-tubule correlation analysis

**Figure 2:**
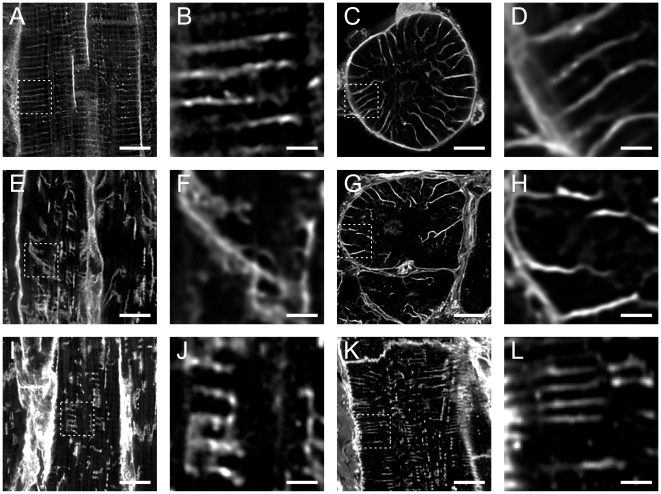
WGA labelling of t-tubules in normal and failing human ventricular myocytes. The top row shows images from normal cells in longitudinal and transverse sections (left to right) and corresponding images from diseased tissue is shown in the lower two rows. (**A**) Longitudinal sections of normal tissue shows uniformly spaced t-tubules. Occasional axial elements can also be seen. (**B**) A magnified view of the region shown by the box in **A**. (**C**) Normal myocyte in transverse section. A radial “spoke-like” organization of t-tubules is apparent. (**D**) Enlarged view of the region shown by the box in **C**. (**E**, **I**, **K**) Longitudinal sections from three different diseased cells, demonstrating the range of t-tubular morphologies found in disease with corresponding (**F**, **J**, **L**) magnified views. Note that while the enlarged view in L appears relatively normal, other regions with the same cell (**K**) are clearly abnormal. (**G**) Transverse section showing that, while the general direction of diseased tubules is radial, tubules are more disorganized. (**H**) Magnified view of the region shown by the box in **G**. Images are projections of 5 slices with z depth of 1 µm. Scale bars in overview images are 10 µm and in close up images 2 µm.

To quantify changes in t-tubule orientation, a 10×10 µm region of WGA labelled sections were taken perpendicular to the plasma membrane and z-projected over 4 image planes (0.8 µm). The analysis involved rotating the images in a clockwise direction in 1° increments and calculation of the autocorrelation. The autocorrelation was then cross-correlated with a line (1 pixel wide) to give the amount of signal in the direction set by the image rotation. To test this analysis, two model t-tubule images were created and correlated to the reference image, one with tubules running at 90° angle (Figure 2A) and the other with tubules running at 45° angle (Figure 2B).

### Image deconvolution

The PSF of the microscope was determined using 100 nm fluorescent latex beads (Molecular Probes - Invitrogen, NZ) in the mounting medium. Iterative constrained deconvolution was performed using the Richardson-Lucy maximum likelihood algorithm as described elsewhere [26].

### RyR and f-actin analysis

To quantify the volume of the cell occupied by contractile apparatus an 8 µm×8 µm×4 µm volume of *f-actin* labelling was converted into binary mask using an automatic thresholding algorithm [27]. The volume of the binary mask was then determine and expressed as percentage of labelled pixels over total. The number of RyR clusters were then measured for the same volume by using a detection algorithm [9] and expressed per unit volume of *f-actin*.

### RyR and DHPR colocalization

The amount of RyR and DHPR colocalization was determined in 8 µm×8 µm×2.5 µm volumes using Pearson correlation coefficient and Manders coefficients for each label [28]. For the calculation of Manders coefficients an automated threshold detection was used [27]. To provide an estimate of the actual overlap between DHPR and RyR signals along the t-tubule, the intensity plot of RyR and DHPR labelling were measured along WGA labelled tubule. A mask of RyR labelling was created by determining full width half max of RyR intensity peaks. DHPR labelling within the RyR mask was considered colocalised.

### Statistical analysis

Data was expressed as mean and standard error or median and interquartile range for non-parametric data. Differences between normal and diseased cells were tested with Students t-test or the Mann-Whitney U test where data did not have equality of variance as determined by Levene's test. *P*<0.05 was considered statistically significant.

## Results

### WGA labelling of normal and failing human cardiac myocytes

WGA was used to visualize t-tubules of ventricular myocytes since it binds to sialic acid residues of the cell membrane glycocalyx [29]. The longitudinal section of healthy heart (Figures 2A and 2B) shows a regular spacing of parallel t-tubules that run approximately perpendicular to the cell surface, although there are t-tubules that bifurcate to connect adjacent z-lines. In transverse sections of healthy myocytes (Figures 2C and 2D) the t-tubules run in a radial direction giving rise to a “spoke-like” appearance, however the t-tubules tend to curve as they approach the centre of the cell. In longitudinal sections of failing heart tissue, there is a marked reduction in the degree of order within the t-system (Figures 2E and 2F). In diseased cells, t-tubules are often seen to run in an oblique direction and can cross several sarcomeres. In diseased cells there is also a less uniform distribution of tubules with axial tubules becoming more common. In transverse sections of diseased cells (Figures 2G and 2H), the direction of the t-tubule was generally toward the cell centre in a spoke-like pattern but appears more disjointed and tortuous.

**Figure 3:**
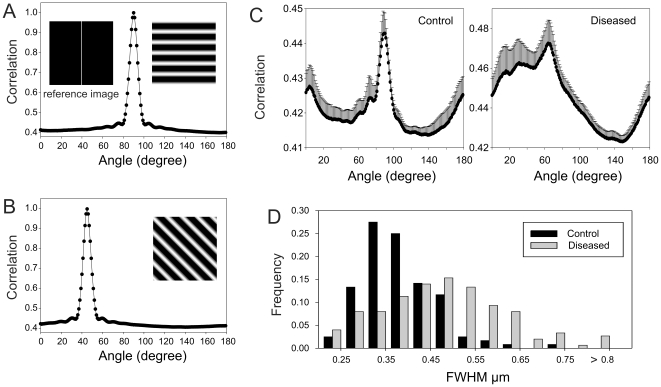
Analysis of changes in ventricular myocyte t-tubule geometry in human heart failure. Correlation analysis of t-tubule angle: To illustrate the analysis, two model t-tubule images were created and correlated to the reference image, one with tubules running at 90° angle (panel **A**) and the other with tubules running at 45° angle (panel **B**), demonstrating that the angle with peak correlation intensity matches the direction of tubules in the model images. (**C**) Correlation analysis of longitudinal sections to measure t-tubule orientation referred to the cell surface. Analyses of normal cells are shown in the left panel and diseased tissue are shown on the right. Note the change in predominant tubule angle and increased spread of t-tubule angles in the diseased tissue. (**D**) Full width at half maximum (FWHM) measurements of control and diseased tubules corrected for optical blurring. In normal myocytes, tubule widths are smaller and have a narrower size distribution compared to diseased myocytes (p = 0.0003 see text).

A correlation method was used to determine the distribution of angles made by WGA labelled t-tubules relative to the cell membrane (see Methods for further details). As shown in Figure 3A and 3B, the analysis could detect the predominant angle of tubular structures. Figure 3C shows that the median angle of t-tubules relative to the cell surface was 95±8° in normal cells (n = 12 cells from 4 hearts), indicating that in normal myocytes the t-tubules are organized in a radial fashion, supporting the visual impression gained from Figure 2. In diseased tissue, the alteration in t-tubule organization resulted in an increase in the width of the distribution of t-tubule angles and the median angle was reduced to 57±9° (P<0.001, Mann-Whitney U, n = 18 cells from 6 hearts). In addition, there was a much greater variability in the t-tubule direction as seen by the marked increase in the width of the distribution of t-tubule angles.

T-tubule diameters were measured by the full width at half maximum (FWHM) of the intensity profile across their images. The size distribution of 120 control t-tubules (from 12 control cells and 4 hearts) and 150 diseased tubules (from 15 cells and 5 hearts) are shown in Figure 3D. The mean diameter of WGA labelled t-tubules of diseased cells was 0.47±0.02 µm (n = 15) and was significantly larger (P<0.001, Mann-Whitney U) than the mean control tubule size of 0.38±0.01 µm (n = 12). Although close to the diffraction limit, these values are larger than reported for rat ventricular myocytes [26] but similar to those reported for rabbit [30]. In addition, the variance of t-tubule diameters was larger in the diseased tissue (P<0.001, Levene's test) so that the change in t-tubule diameter was not simply due to an increase in diameter of all tubules. These data show that diseased myocytes can undergo significant remodelling of t-tubular structure with a loss of radial directed t-tubules and an increase in oblique and longitudinally oriented t-tubules. In addition, a wide variation is t-tubule morphology is apparent with some t-tubules appearing almost normal while others flatten, fragment and/or become more filigree in appearance (even within a single diseased cell).

### T-tubules without WGA labeling

**Figure 4:**
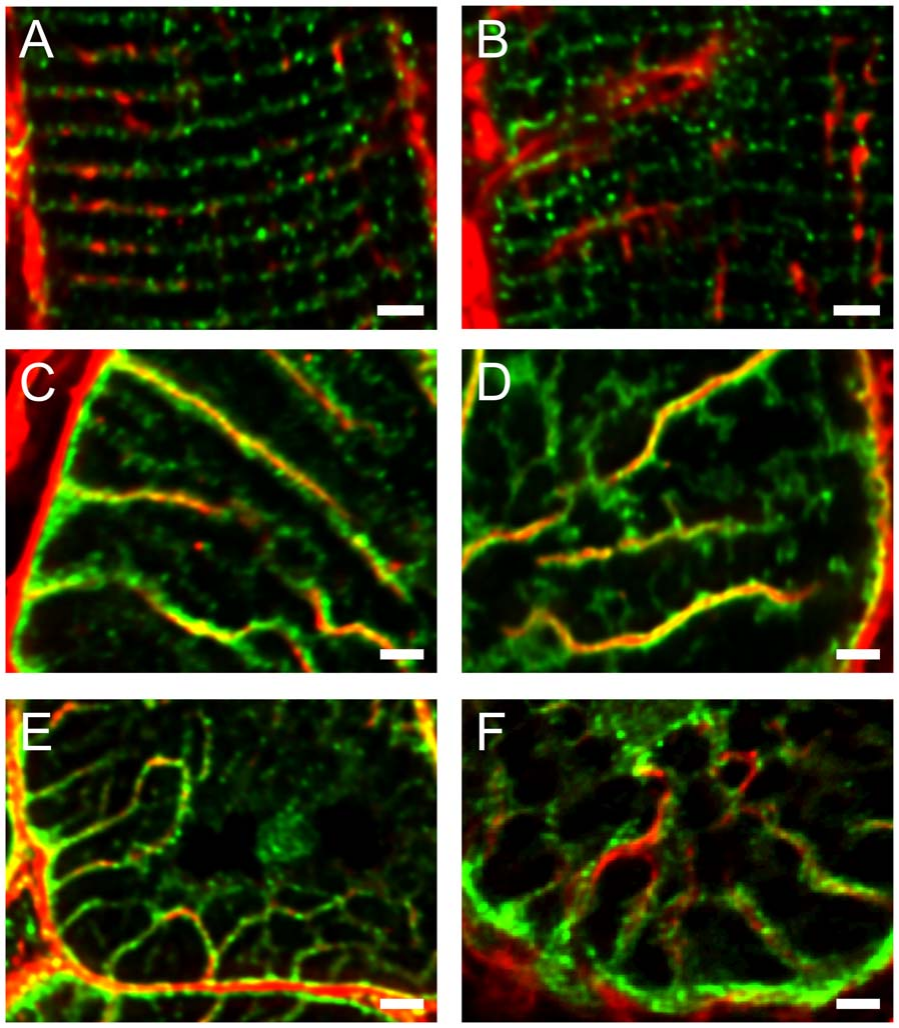
Sarcolemmal protein labelling with WGA in normal and failing human cardiac myocytes. Panels are arranged so that the left column corresponds to normal and the right column diseased tissue. **A** and **B** show longitudinal sections of control and diseased myocytes respectively, labelled for DHPRs (green) and WGA (red). The DHPR labelling is more continuous across the cell than the WGA label. Panels **C** and **D** show transverse images labelled for NCX (green) and WGA (red). In control myocytes (**C**), finer tubules containing only the NCX label can be seen to connect to larger spoke like tubules containing both labels. Diseased (**D**) myocytes have similar labelling pattern but many connections between larger tubules appear broken. Panels **E** and **F** show transverse sections labelled for Cav3 (green) and WGA (red). (**E**) Again, finer tubules labelled with only Cav3 connect larger tubules containing both labels. Diseased myocytes (**F**) have similar labelling pattern but with a reduction in fine Cav3 labelling between larger tubules. Images are z projections of 4 slices with z depth of 0.8 µm from deconvolved image stacks. Scale bars are 2 µm.

**Figure 5:**
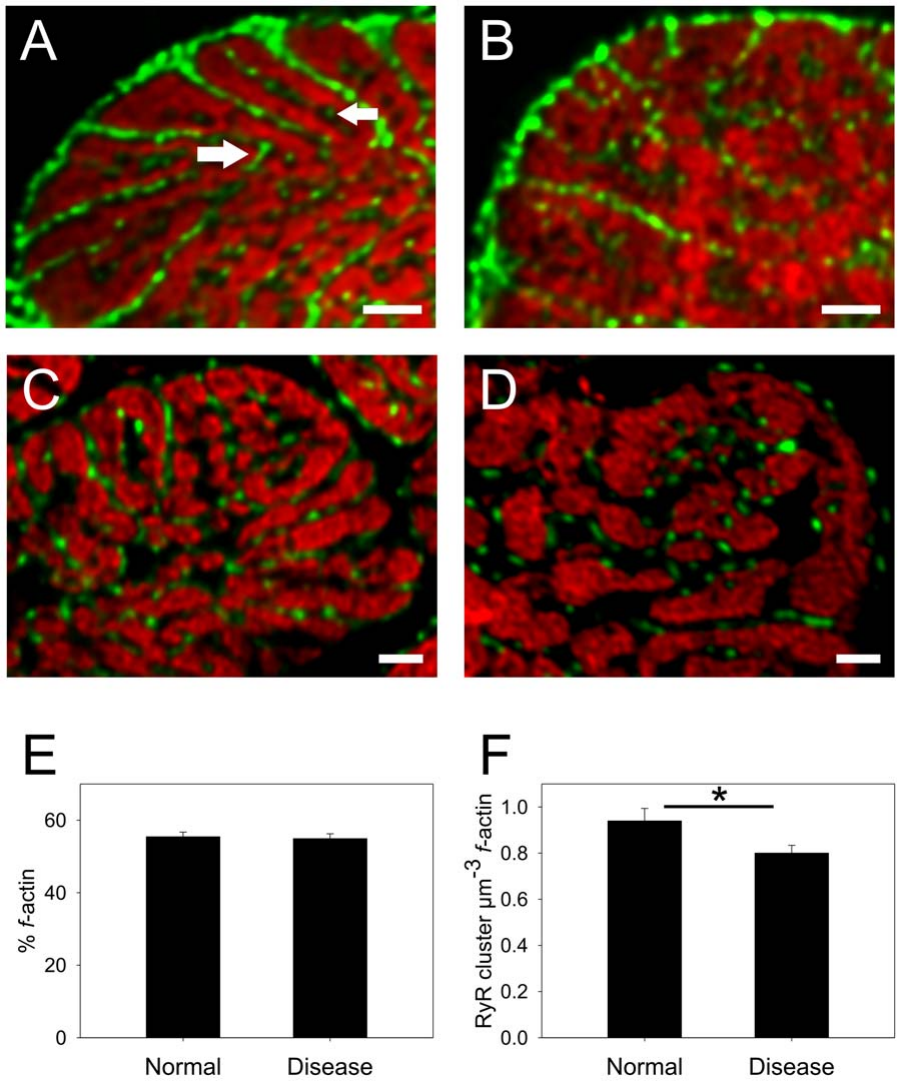
Comparison of myofilaments, t-tubules and RyR localization. Panels **A** and **B** show transverse sections of normal and diseased myocytes respectively, labelled for Cav3 and *f*-actin. The contractile machinery is surrounded by Cav3 labelling at this plane which was selected to be centred at the z-line. Gaps in the centre of contractile bundles contain isolated Cav3 labelling (small white arrow) and inspection of adjacent sections show that this arises from axial tubules that join transverse tubules outside the presented image plane. A connection between adjacent radial t-tubules is indicated by the large arrow. Panels **C** and **D** show transverse sections of normal and diseased tissue (respectively) labelled for RyR and *f*-actin. It is apparent that each contractile bundle is surrounded by several RyR clusters. All images are projections of 4 slices with z depth of 0.8 µm. Panel **E** shows analysis of cell volume occupied by *f*-actin and no significant difference (P = 0.81) exists between normal and diseased tissue. Panel F shows analysis of the number of RyR clusters per µm^3^ of *f*-actin and there was a significant reduction in the density of RyR clusters in disease cells (*P = 0.03). Scale bars are 2 µm.

There appeared to be large regions of the cell without obvious t-tubule labelling but without a suitable lipid marker (for fixed tissue) it is possible that some t-tubules might not be WGA labelled due to a change in glycosylation and therefore WGA labelling. To examine this possibility, tissue from 3 control and 3 diseased hearts were dual labelled for WGA and various sarcolemmal membrane proteins: dihydropyridine receptors (DHPR), sodium calcium exchangers (NCX), and caveolin 3 (Cav3). This labelling suggested that the t-system was more extensive than indicated by WGA alone (see Figure 4). For all labels examined, the apparently wider t-tubules, which contained strong WGA labelling, are extended and connected by thinner tubules which contain DHPR, NCX and Cav3. These thinner tubules formed ring-like structures which would be consistent with these tubules surrounding myofibrils (see Figure 5). In diseased myocytes, similar labelling patterns are seen but the organization of the thinner tubules was generally disrupted.

### Relationship of t-tubular network to myofibrils

Since Cav3 provided a reliable marker of surface membrane, we compared the distribution of Cav3 labelling to that of myofibrillar *f*-actin (Figure 5A and 5B). Myofibrils generally followed the spoke-like arrangement of t-tubules so the contractile machinery appeared as flattened wedges or plates in cross section. In places, Cav3 labeled t-tubules formed anastamoses between the radial t-system spokes (large arrow) and some isolated Cav3 labeling could be seen surrounded by myofibrils (small arrow). Examination of adjacent sections (not shown) showed that these tubules were axially oriented and joined radial t-tubules at the next z-line. A similar relationship between Cav3 and *f-*actin was seen in diseased cells (Figure 5B) but the overall pattern of labelling was more disjointed with the wedge-like pattern of contractile machinery becoming less obvious.

### Relationship between ryanodine receptors and contractile machinery

Sections were labelled for RyR and *f-*actin (Figure 5C and 5D) and analysed in three dimensions (3D). This labelling showed that each myofilament bundle was surrounded by several RyR clusters and there was no obvious change in the labelling between control and diseased cells (beyond a subtle reorganization of the myofibrils). To assess the volume of the cell occupied by contractile apparatus, a binary mask of *f-*actin labelling was created and expressed as percentage of total area. This analysis (Figure 5E) showed no significant change (P = 0.81, Student's t-test) in the fraction of cell volume occupied by the contractile apparatus in control myocytes (55±1%, n = 9 cells, 3 hearts) compared to diseased myocytes (55±1%, n = 18 cells, 6 hearts). The number of RyR clusters in the analysis volume was counted using a cluster detection algorithm [9] which showed a small but significant (P = 0.03, Student's t-test) reduction in the number of RyR clusters per unit volume of contractile apparatus in diseased myocytes (Figure 5F). In control, there were approximately 0.94 clusters per µm^3^ *f*-actin which would equate to a cell wide cluster density of 0.52 RyR clusters per µm^3^ cell volume (given 55% of cell volume occupied by myofilaments).

### RyR and DHPR labeling

**Figure 6:**
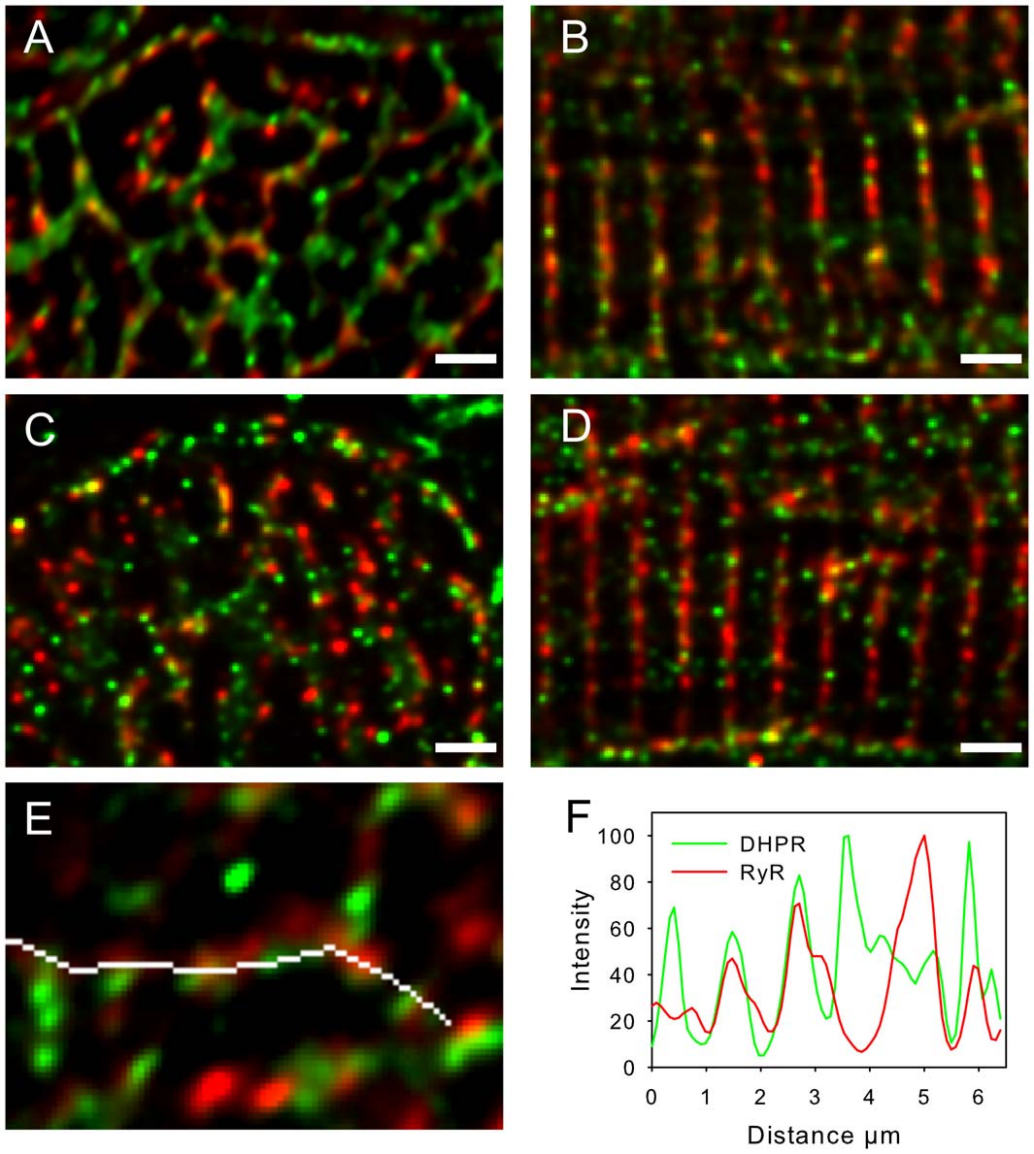
RyR and DHPR cluster colocalization is reduced in failing human cardiac myocytes. Panels **A** and **B** show images of control myocytes in transverse and longitudinal orientation respectively, labelled for RyR (red) and DHPR (green). Panels **C** and **D** show images of diseased myocytes in transverse and longitudinal orientation respectively, labelled for RyR (red) and DHPR (green). Images are z projections of 4 optical sections with z depth of 1 µm. Scale bars are 2 µm. Panel **E** shows an exemplar region of DHPR and RyR labelling in a normal cell, this image is a single optical section. The white line indicates the position of intensity readings along a WGA labelled t-tubule (label not shown for clarity). Panel **F** shows the intensity profile of DHPR and RyR labelling along the white line shown in panel **E**. Similar results were seen with an alternative DHPR antibody (see methods and supplementary Figure S1).

Figure 6 shows tissue dual labeled for DHPRs and RyRs. The RyR labelling was more punctate than the DHPR labelling and in longitudinally orientated normal myocytes, DHPR labelling occurred predominantly at the Z-line. In diseased cells, some DHPR label was displaced from the Z-line. This visual observation was supported by colocalisation analysis by Pearson's and Mander's coefficients (in 3D) which showed a significant reduction in DHPR colocalization (Table 1). This may reflect the axial distortion of the t-system in diseased myocytes as shown above and/or the reduction in number of RyR clusters shown in Figure 5F.

**Table 1:** Colocalisation analysis of DHPR and RyR labeling.

Tissue	Cell orientation	number	Pearson's correlation		DHPR colocalisation(Manders)		RyR colocalisation(Manders)	
Normal	Longitudinal	9	0.33±0.03	*P = 0.001	0.46±0.03	*P<0.001	0.24±0.03	P = 0.09
Diseased	Longitudinal	18	0.22±0.01		0.24±0.02		0.18±0.02	
Normal	Transverse	9	0.33±0.06	P = 0.72	0.26±0.03	P = 0.34	0.30±0.02	P = 0.14
Diseased	Transverse	18	0.32±0.03		0.37±0.05		0.29±0.03	

*Significantly different at p<0.05.

The distribution of RyR and DHPR labels along a WGA-labelled t-tubule from a normal myocyte is illustrated in Figure 6E and 6F (see also Supplementary Figure S1). Both labels were non-uniform, and a density plot (Figure 6F) shows that local concentrations of DHPR were not perfectly aligned with the RyR clusters although considerable overlap between both labels was present. To quantify these data, we constructed a mask by thresholding the RyR label at 50% maximum local intensity and integrated the fraction of DHPR label signal within the mask boundaries. In these transverse sections, we found that 51±3% (9 t-tubules from 9 cells) of the DHPR labelling was within the region defined by RyR labeling, which was not significantly different in disease (50±4%, p = 0.81), in agreement with the co-localization measured by Pearson's and Manders coefficients (Table 1). Given the smaller fraction of membrane area occupied by jSR (e.g. ∼21% in rabbit [31]), suggests that there was a preferential localization of DHPRs near RyRs, albeit less pronounced than in other species (see Discussion). Labelling with an alternative antibody directed toward the N-terminus of the DHPR showed similar labeling patterns (see Supplementary Figure S1).

## Discussion

This report presents a detailed analysis of the architecture of cellular structures critical to excitation-contraction coupling in normal and failing human myocardium.

Quantitative analysis of WGA labelled t-tubules in ventricular myocytes demonstrated major alterations in the tubular network in diseased cells compared to normal cardiac myocytes. Although some changes in the pattern of WGA labelling have been previously noted [21] these changes have not been quantified. An unexpected finding was that WGA labelled only a portion of the t-system as labels for the sarcolemmal membrane proteins DHPR, NCX and Cav3 revealed a more extensive network of fine tubules. Disruption of t-tubule architecture in diseased cells was associated with a small loss of RyR clusters and reduced colocalization between RyRs and DHPRs. This indicates remodelling of the t-tubular network in the failing human heart may contribute to calcium handling abnormalities seen in heart failure and underpins many functional studies in animal models.

### Structure of the human t-system

Two previous studies that have examined the t-system in human cardiac myocytes, although not at the level of detail shown here. One of the papers described major changes [24] while no major changes were found in a second study [25] Our results show that major changes in the structure of t-tubules can occur in human heart failure, in general agreement with conclusions from animal models of heart failure. However, it is important to note that there is a greater variation in t-tubule pathology seen in human myocytes (see Figure 2) than the generally simpler loss of t-tubules described for animal models. We suspect that the high degree of t-tubule morphology shown here may reflect the long(er) duration of the disease process in humans. Our results also largely agree with previous immunohistochemical examination of cytoskeletal proteins [22], [23], [32], [33], [34]. A potential explanation for the lack of changes reported by Ohler *et-al* (2009) [25] might be arise from selection bias associated with myocyte isolation, which would be expected to “purify” healthier myocytes. We have found that quite normal t-tubular structure can be found adjacent to severely disrupted t-tubules.

Importantly, our results show that WGA labels only a portion of the t-tubular network in human myocytes since DHPRs, NCX and Cav3 labels revealed an additional fine network of sub-resolution structures that connected the larger WGA-labelled t-tubules (Figure 4). This indicates that the t-system in humans is more extensive than might be deduced from WGA labelling alone. This is a noticeable difference between human and animal cells, as, for example there is a high degree of overlap between Cav3 and WGA labeling in rat myocytes [35]. Although the cause and role of the changes in glycocalyx as reported here within t-tubules is unclear, it is possible that the expansion of the glycocalyx drives the remodelling of parts of the t-tubule network. It is also possible that some hypertrophic signals, which lead to the expansion of cytoskeletal proteins [36] in heart failure, also drive t-system remodelling.

In contrast to our results, Kaprielian et al. [32] suggested there was a proliferation of t-tubules in diseased myocytes but the extent of this proliferation was not quantified. However, we suggest that their different conclusion may have arisen from the dilation of t-tubules in disease reported here which would have made them more obvious. In connection with this point, Figure 8D in their paper showed a dilated non-radial t-tubule in EM similar to the observations reported here. We conclude that our results are more in line with studies of the t-system in animal models and humans which have described either a loss and/or disorganisation of the t-system [10], [11], [21], [24], [37] rather than an expansion. It should also be noted that, even in normal tissue, the general architecture of the human t-system is different (i.e. predominantly spoke-like) to the t-system rete described for rat [26] and we did not detect frequent varicosities along the length of t-tubules as recently described for rabbit [30].

### T-tubule disorganisation and E-C coupling proteins

In addition to a marked reorganisation of the t-system within the failing human heart, we found a small reduction in the number of RyR clusters per unit myofibril area and a decrease in colocalization between DHPRs and RyRs. The importance of the local geometric arrangement of DHPRs and RyRs has been highlighted in modelling studies where EC coupling gain is diminished with small distortions in junctional geometry [12]. Our results support this idea, although the level of colocalization observed between DHPR and RyR clusters in control human ventricular myocytes suggests that DHPRs are not almost exclusively restricted to the couplon as has been suggested to be the case in rat [38]. Nevertheless on average, approximately 51% of the DHPR label was closely associated with the RyR clusters so that these data still support the idea of tight regulation of RyRs by DHPRs. Our analysis of RyR and DHPR longitudinal colocalization demonstrated an apparent weakening of the overlap between DHPR and RyR clusters in failing human heart cells. That such a change was not observed in transverse section may be explained by the poorer axial resolution of the microscope and the change in t-tubule orientation towards a axial direction. Even if the couplons themselves did not move away from z-lines, the presence of non junctional DHPRs in displaced t-tubules would still lead to a reduction in colocalisation. In a rat model of heart failure, Song *et. al* [14] reported a similar (∼20%) loss of colocalization between RyRs and DHPRs coupled with t-tubule remodelling and “dysynchronous” calcium spark formation. This effective displacement of mean DHPR location from RyRs would result in a reduced ability of the Ca^2+^ current to activate SR release and therefore a loss of E-C coupling “gain” [3], [13] While we were unable to measure the functional consequences of the changes in key EC coupling proteins/structures measured here, the possibility that the failing human myocyte may suffer reduced EC coupling fidelity seems very likely. However, it should also be noted that the spatial reorganization of the t-tubule network in human heart failure that we report here is quite different to the loss of transverse tubules and an increase axial tubules reported for the SHR model [14]. We suggest that future study of the mechanisms underlying the control of t-tubule remodelling may lead to new directions for treating heart failure.

## Supporting Information

Figure S1 **DHPR immunohistochemistry with an alternative antibody gives generally similar DHPR labelling patterns relative to RyR.** This antibody was raised against a peptide corresponding to residues 1–46 of rabbit Cav1.2a N terminus (ACC-013, Alomone). Panels **A** and **B** show longitudinal images of control and diseased myocytes respectively, labelled for RyR (red) and DHPR (green). Images are z projections of 4 optical sections with z depth of 1 µm. Scale bars are 2 µm. It is apparent that RyR and DHPR labelling is non-uniform with only moderate colocalisation. In disease cells greater proportion of DHPR appears between Z-lines. The white line indicates the position of intensity readings along a WGA (blue) labelled t-tubule. Panel C shows the WGA labeling (blue) together with RyR (red) and DHPR (green) in a single transverse section. As shown for the other antibody (ACC-003) in the manuscript, there was no significant difference in the measured overlap between RyR and DHPR labelling (as a fraction of total DHPR labeling) between normal and diseased samples (46%±7 vs. 55%±4 respectively n = 6 in each group, p = 0.46). Panel **D** shows an intensity profile of DHPR and RyR labelling along the white line following the t-tubule shown in panel **C**.
(TIF)
