# Correction: Changes in the Organization of Excitation-Contraction Coupling Structures in Failing Human Heart

**DOI:** 10.1371/annotation/061613ea-0f01-420f-bc3f-af36e5c35790

**Published:** 2011-04-26

**Authors:** David J. Crossman, Peter R. Ruygrok, Christian Soeller, Mark B. Cannell

The middle initial of the second author is incorrect. The correct spelling is: Peter N. Ruygrok.

The correct citation is: Crossman DJ, Ruygrok PN, Soeller C, Cannell MB (2011) Changes in the Organization of Excitation-Contraction Coupling Structures in Failing Human Heart. PLoS ONE 6(3): e17901. doi:10.1371/journal.pone.0017901

The correct author contributions are: Conceived and designed the experiments: MBC DJC PNR. Performed the experiments: DJC. Analyzed the data: MBC DJC CS. Contributed reagents/materials/analysis tools: MBC CS PNR. Wrote the paper: DJC MBC. 

