# Supplementary figures and images for: Changes in the Organization of Excitation-Contraction Coupling Structures in Failing Human Heart
Source: PLoS One. 2011 Mar 9;6(3):e17901. doi: 10.1371/journal.pone.0017901 (PMC3052389; doi:10.1371/journal.pone.0017901)

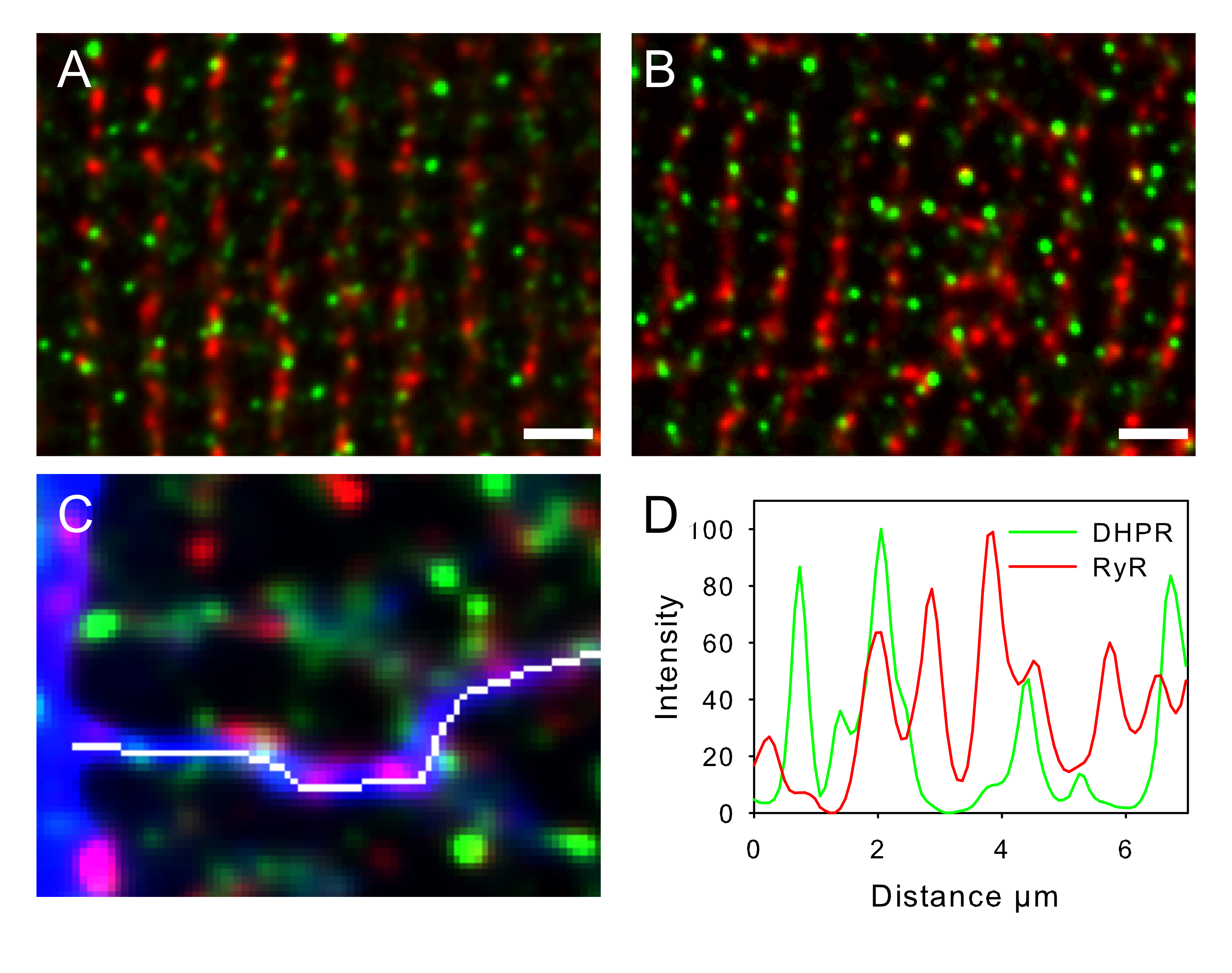

Supplement: Figure S1 — DHPR immunohistochemistry with an alternative antibody gives generally similar DHPR labelling patterns relative to RyR. This antibody was raised against a peptide corresponding to residues 1–46 of rabbit Cav1.2a N terminus (ACC-013, Alomone). Panels A and B show longitudinal images of control and diseased myocytes respectively, labelled for RyR (red) and DHPR (green). Images are z projections of 4 optical sections with z depth of 1 µm. Scale bars are 2 µm. It is apparent that RyR and DHPR labelling is non-uniform with only moderate colocalisation. In disease cells greater proportion of DHPR appears between Z-lines. The white line indicates the position of intensity readings along a WGA (blue) labelled t-tubule. Panel C shows the WGA labeling (blue) together with RyR (red) and DHPR (green) in a single transverse section. As shown for the other antibody (ACC-003) in the manuscript, there was no significant difference in the measured overlap between RyR and DHPR labelling (as a fraction of total DHPR labeling) between normal and diseased samples (46%±7 vs. 55%±4 respectively n = 6 in each group, p = 0.46). Panel D shows an intensity profile of DHPR and RyR labelling along the white line following the t-tubule shown in panel C. (TIF) [file pone.0017901.s001.tif]
